# Women’s empowerment in agriculture and agricultural productivity: Evidence from rural maize farmer households in western Kenya

**DOI:** 10.1371/journal.pone.0197995

**Published:** 2018-05-31

**Authors:** Gracious M. Diiro, Greg Seymour, Menale Kassie, Geoffrey Muricho, Beatrice Wambui Muriithi

**Affiliations:** 1 International Center for Insect Physiology and Ecology (icipe), Nairobi, Kenya; 2 CGIAR Research Program on Policies, Institutions, and Markets – International Food Policy Research Institute, Washington, DC, United States of America; Kansas State University, UNITED STATES

## Abstract

This paper documents a positive relationship between maize productivity in western Kenya and women’s empowerment in agriculture, measured using indicators derived from the abbreviated version of the Women’s Empowerment in Agriculture Index. Applying a cross-sectional instrumental-variable regression method to a data set of 707 maize farm households from western Kenya, we find that women’s empowerment in agriculture significantly increases maize productivity. Although all indicators of women’s empowerment significantly increase productivity, there is no significant association between the women’s workload (amount of time spent working) and maize productivity. Furthermore, the results show heterogenous effects with respect to women’s empowerment on maize productivity for farm plots managed jointly by a male and female and plots managed individually by only a male or female. More specifically, the results suggest that female- and male-managed plots experience significant improvements in productivity when the women who tend them are empowered. These findings provide evidence that women’s empowerment contributes not only to reducing the gender gap in agricultural productivity, but also to improving, specifically, productivity from farms managed by women. Thus, rural development interventions in Kenya that aim to increase agricultural productivity—and, by extension, improve food security and reduce poverty—could achieve greater impact by integrating women’s empowerment into existing and future projects.

## 1. Introduction

In many economies in sub-Saharan Africa (SSA), women provide most of the labour force for agricultural production [1, 2, 3]. In Kenya, for example, women make up between 42% and 65% of the agricultural labour force [4, 5], in addition to their traditional domestic responsibilities. Despite women’s important role in the agricultural sector, however, empirical evidence shows that they lag behind men with regard to agricultural productivity in SSA due to the gender inequalities that persist in respect of access to, control over and utilisation of productive resources such as land, livestock, labour, education, extension and financial services, and technology [4, 6, 7, 8, 9, 10, 11,12,13,14,15, 16]. Inequality in landholding is especially severe in SSA. Cultural norms and traditions restrict women’s ability to inherit land [17, 18] and contribute to widening gender gaps in the quality and size of owned farmland [19]. In Kenya, a mere 0.5% of women have access to financial services [20] and only around 6% own land [21]. Limited land ownership by female farmers hinders their access to formal credit, since land is a major form of collateral [22]. Other gender inequalities evident for women in Kenya’s agricultural sector include limited access to labour and agricultural markets [23, 24] and less control over revenue from agricultural production than men [25, 26]. In addition, women farmers in Kenya spend more time in care and domestic work than their male counterparts [2, 24], which can limit their access to productive resources such as extension and advisory services [27] and participation in income-generating activities [24].

Reducing gender inequality is widely recognised around the world as contributing to agricultural growth and the attainment of food and nutritional security [1, 5]. More specifically, many international development programmes, such as the United States Agency for International Development (USAID) Feed the Future initiative, which also operates in Kenya, perceive women’s empowerment as a key factor in closing gender gaps in agricultural productivity [28, 29, 30, 31, 24, 6]. Research has also shown that empowering women can lead to improvements in their status both inside and outside the household—including greater control over household resources; better mental health; reduced time constraints; and increased access to financial services, health care, skills development, income-earning opportunities, information about markets and legal rights—all of which may, in turn, positively impact agricultural productivity, nutrition and food security [6, 32, 33, 34]. Hence, promoting gender equality is a major focus of rural development policy that aims to achieve sustained food security and poverty alleviation in agrarian economies, including those in SSA [35, 36, 37]. With specific respect to SSA, therefore, understanding the role of women’s empowerment in agriculture is important for policymakers and development partners interested in devising more effective interventions to increase agricultural productivity, enhance household and national economic growth, achieve food security, improve nutrition, and reduce poverty.

In this paper, using smallholder maize farmers in western Kenya as a case study, we examine the effects of women’s empowerment in agriculture on maize productivity at farm- and plot-level, where women’s empowerment may have differential effects on such productivity depending on whether a plot is managed jointly by a man and a woman, or individually by either a man or a woman. We contribute to the existing literature on gender and agricultural productivity by using a more comprehensive measure of women’s empowerment in the agricultural sector compared to previous studies: the (abbreviated version of the) Women’s Empowerment in Agriculture Index (WEAI) [28]. Whereas the original WEAI utilised ten component indicators, the Abbreviated WEAI (A-WEAI) comprises only six. Like its predecessor, the A-WEAI measures women’s empowerment in their roles and extent of engagement across five agricultural domains (production, resources, income, leadership and time) based on individual-level survey data [28, 38]. Each dichotomous indicator measures whether an individual respondent has achieved adequacy, based on the definitions shown in Table 1, with corresponding weights that ensure that each domain receives equal weight when the indicators are aggregated together. We operationalise the A-WEAI in two ways. First, we determine a woman’s aggregate achievement of empowerment (i.e., her overall empowerment score) across the six weighted indicators (see Table 1 for indicator descriptions and weights). Second, we consider a woman’s level of empowerment in terms of each individual A-WEAI indicator. A woman is defined as “empowered” if she has achieved adequacy in at least 80% of the weighted indicators (equivalent to four out of the five domains) [28].

**Table 1:** Description of domains and empowerment indicators in the abbreviated women’s empowerment in Agriculture Index.

Domain	Indicator	Definition of *adequacy* (= 1)	Weight
Production	Input in productive decisions	Sole or joint participation in *at least one* decision related to food and cash-crop farming, livestock farming, and fishery production	1/5
Resources	Asset ownership	Sole or joint ownership of *at least one* major household asset	2/15
	Access to and decisions on credit	Sole or joint control or participation in decision-making on credit from *at least one* source	1/15
Income	Control over use of income	Sole or joint control over income for *at least one* of food and cash-crop farming, livestock farming, and fishery production	1/5
Leadership	Group membership	Active member in *at least one* formal or informal group	1/5
Time	Workload	Spent *less than or equal to 10*.*5 hours* on paid and unpaid work during the previous day	1/5

Source: [38]

Although some empirical studies have used the WEAI to investigate the impact of women’s empowerment on food security and nutrition-related outcomes [e.g. 6, 33, 34, 38, 39, 40, and 41], and have generally found that women’s empowerment has the potential to improve both such outcomes, thus far only a single study [42] has used the WEAI to explore what effects women’s empowerment may have on agricultural productivity. Other related studies have mainly focused on whether and how farm productivity is affected when inputs are redistributed between women and men instead of only among men in households (e.g., [12, 43, 44, 45, 46]). However, the findings from such studies may be less useful for effective policy prescription because they do not completely account for women’s roles or the extent of women’s engagement in the agricultural sector—a limitation that the WEAI overcomes through differentiating among the five domains listed above.

The key finding from our study is that women’s empowerment in Kenya agriculture can spur increased maize productivity among smallholder farmer households. Furthermore, whereas all women’s empowerment indicators (except *workload*) significantly increased productivity, the number of production decisions indicators seem to have greatest effect on productivity. The results further show that, female- and male- managed plots experienced significant improvements in productivity when the women who tended them were more empowered. These results suggest that future rural development interventions that aim to increase agricultural productivity in Kenya could achieve greater impact by integrating women’s empowerment into existing and future projects, e.g., by focusing on women’s access to credit, asset accumulation and community leadership.

The conceptual model and estimation methods adopted in the study are presented in the next section, which is followed by a description of the data and of selected characteristics of the sample households. The fourth section presents and discusses the estimation results, while the fifth and final section concludes the presentation of the study with a discussion of its policy implications.

## 2. Conceptual model and estimation methods

### 2.1 Model specification

We conceptualise the relationship between women’s empowerment and agricultural productivity in terms of a collective model of intra-household bargaining in which households are considered as a collection of discrete individuals, each with his/her own set of preferences, rather than as a single, monolithic decision-making unit [47, 48]. The chosen framework explicitly allows for intra-household differences in preferences and heterogeneity in the impact of bargaining power on household members’ ability to negotiate and allocate resources to maximise their respective utilities [36]. Our selection of the collective model was based on the large body of empirical literature (e.g. [49, 50, 51]) that has demonstrated the inaccurate approximation of intra-household behaviour via the traditional unitary model [52]. For example, studies have shown that the relative bargaining power of women and men within a household largely depends on their relative access to, control over and utilisation of resources [24]. Such relative power may also directly influence agricultural productivity in a household through its effect on household members’ ability to allocate and organise productive resources optimally [53, 54].

Within this framework, plot-level productivity gains from women’s empowerment might be expected to differ, depending on which household member manages the plot in question. While the productivity gains from women’s empowerment are expected to be strongest for plots cultivated either solely or jointly by women, it is also possible that the productivity-enhancing effects of women’s empowerment extend (“spill over”) to plots operated by others within the household, e.g., via the sharing of information, pooling of resources, or positive peer pressure. Indeed, a recent study [42] using WEAI data from Bangladesh found that improvements in women’s empowerment were associated with higher levels of technical efficiency on all plots cultivated by a household, regardless of whether a woman actively managed the plot or not.

We examine the relationship between women’s empowerment and farm productivity by extending the standard productivity or yield function to include a measure of women’s empowerment as an additional input in the household maize production function, and we test whether the impact of women’s empowerment on yields differs for plots managed jointly by the man and the woman in a household, and individually by either of them. Thus, the maize yield of household *i* from plot *p* is as specified in Eq 1:

$$Q_{ip}\;=\;f(W_{i},X_{i},K_{ip},P_{ip})$$ (1)

In this equation, *Q*_*ip*_ denotes the quantity of maize yield per acre produced by household *i* from plot *p*; *W*_*i*_ is a measure of women’s empowerment status in agriculture, based on the A-WEAI; *X*_*i*_ is a vector of household- and community-level explanatory variables that influence production decisions; *K*_*ip*_ denotes a vector of inputs used in maize production on plot *p*; and *P*_*ip*_ denotes plot-level attributes of plot *p*.

We acknowledge that women’s empowerment status is potentially endogenous to agricultural productivity. For example, unobserved characteristics such as women’s leadership or farm management skills could potentially affect both household production and their empowerment status. Endogeneity may also arise from reverse causation between women’s empowerment and farm productivity: on the one hand, women’s empowerment may increase agricultural productivity by encouraging a more optimal allocation and organisation of productive resources, while on the other, increased yields may enhance women’s share of farm income, their contribution to household food security, or their status within the community. For example, women from more food-secure households are likely to command more respect in their communities and may be more likely to engage in community leadership activities than their counterparts from less food-secure households. We therefore treat women’s empowerment (*W*_*i*_) as an endogenous variable in the maize yield equation (*Q*_*ip*_) as specified in the following system of equations:

$$Q_{ip}\;=\;\alpha +\psi W_{i}+\gamma K_{ip}+{\lambda P}_{ip}+\varepsilon_{ip}$$ (2)

$$W_{i}\;=\;\pi V_{i}+\delta Z_{i}+u_{i}$$ (3)

where *E*(*u*_*i*_|*Z*_*i*_) = 0 and *E*(*u*_*i*_, *ε*_*ip*_) ≠ 0

In Eq 3, *V* denotes a vector of the explanatory variables, while *Z* denotes a vector of the instrumental variables. The Greek characters denote unknown parameters to be estimated, where *ψ* captures the effect of women’s empowerment in agriculture on maize productivity. The error terms are *ε*_*ip*_ and *u*_*i*_. The other elements are defined as above.

We apply an instrumental variable (IV) method described later in this section to correct for the potential endogeneity of women’s empowerment using the following six variables as instruments: (1) *diversity of associations in village (number of types of association)*, (2) *difference in age between principal male and principal female in household (years)*, (3) *difference in education between principal male and principal female in household (years)*, (4) *whether the woman brought assets into marriage (1 = yes*, *0 = no)*, (5) *years of residence in village (women only)*, and (6) *household composition (by age group)* (see Table 2 for descriptive statistics). We instrument for the overall empowerment score using all the variables except for diversity of associations in the village. For each of the individual empowerment indicators, the specific variables used as instruments vary. Differences in age and education and whether the woman brought assets into her marriage were used as instruments for number of production, asset ownership, income and group membership decisions indicators. Diversity of associations and differences in age and education were used as instruments for number of credit decisions indicator. Differences in age and education and household composition by age category were used as instruments for the workload indicator.

**Table 2:** Instrumental variables.

Variable	Mean	Standard deviation
Diversity of associations in village (number of types of association)	5.84	2.28
Difference in age between principal male and principal female in household	6.79	8.39
Difference in education between principal male and principal female in household (years)	0.36	4.1
Wife brought assets into marriage (1 = yes, 0 = no)	0.14	0.35
Household composition (by age group)		
*• % of household members aged below 5*	0.10	0.13
*• % of household members aged 5–9*	0.12	0.13
*• % of household members aged 10–14*	0.16	0.15
*• % of household members aged 15–19*	0.15	0.15
*• % of household members aged 20–44*	0.24	0.19
*• % of household members aged 45–60*	0.13	0.17
*• % of household members aged above 60*	0.11	0.20
Number of observations	707	

Residing in a village with a high diversity of associations may help women to develop stronger social networks, which might in turn influence their decision to actively participate in such associations. For example, an earlier study [41], which looked at the impact of women’s empowerment on food security in rural Bangladesh, used the number of informal credit sources in a village as an instrumental variable in their analysis. To this same end, our study used a questionnaire to capture what types of association—whether formal or informal—existed in the respondent’s village of residence. These associations were found to comprise informal credit groups, input supply groups, development groups, a marketing group, mutual membership groups, a business association, a water association, a women’s group, civil groups, and a religious group.

As other studies have also shown [50, 34], differences in age and education in respect of a household’s principal decision-makers may reflect differences in human capital and, hence, could indicate women’s relative bargaining position in the household. Similarly, assets brought by a woman into her marriage may also be positively associated with her bargaining position within the household [55, 56, 57]. In our study area, we noted the practice of relatives giving a woman gifts prior to her marriage. Such gifts ranged from smaller items to larger assets such as livestock and farming equipment.

We used the composition of the household by age group to instrument the workload indicator. We grouped household members into one of five categories, as follows: aged less than 5 years, aged between 5 and 9, aged between 10 and 14, aged between 15 and 19, aged between 20 and 44, aged between 45 and 60, and aged above 60. The proportion household members in each of these age categories served as an instrument to how much work and leisure time a woman had in a household. Additional adults of prime working age or adolescent children in a household, particularly other females, may decrease the domestic work burden of the principal woman in the household—if, for example, care-giving duties are shared among household members—whereas each additional dependent (household members aged below 5 or above 60) might increase demand on the principal woman’s time.

The rest of the covariates included in the empirical model were drawn from the empirical literatures on agricultural productivity and women’s empowerment in SSA. These covariates fall into four broad categories: (1) *household socio-economic characteristics*, (2) *agricultural inputs and practices*, (3) *plot-level attributes*, and (4) *community-level variables*. *Household socio-economic characteristics* constitute sex, age, education and livestock ownership. *Agricultural inputs and practices* constitute the quantity of fertiliser per acre, other input expenditure per acre (e.g. seed and agrochemicals), labour input (in person-days/acre), use of yield-enhancing practices (e.g. intercropping, crop rotation, and push—pull technology/PPT), a dummy variable for farmer confidence in the quality of agricultural extension and advisory service provision, and a dummy variable for perceived credit constraints in the household (equal to 1 if a household needed credit but were unable to get it, and 0 otherwise). Push-pul technology (PPT) is a cropping system in which cereals such as maize are intercropped with perennial fodder legumes (*Desmodium*) that repel (‘push’) stemborers and suppress *Striga* species (witchweed). The cereal crops are also surrounded by a border of perennial fodder grass (e.g. *Pennisetum purpureum*/Napier grass or *Brachiaria* species) that attracts (‘pulls’) stemborers away from cereal plants [58]. *Plot-level attributes* constitute the proximity of the household to the farm plot, plot tenure, perceived soil depth (shallow, medium or deep), soil fertility (low, medium or high), slope (gentle, medium or steep), and whether the plot is vulnerable to insect pests and diseases. *Community-level variables* constitute the distance from the household to the nearest input supply shop, output market and extension offices, and sub-region dummies to control for location-specific effects such as culture and unobserved agroecological attributes. Lastly, we also included a season dummy to capture the effects of seasonal weather variation on maize productivity.

### 2.2 Estimation methods

We estimate two alternative specifications of the yield function. In the first, women’s empowerment is operationalised in aggregate, to understand the general maize productivity effect of women’s empowerment. More specifically, following [28], we compute the female respondent’s individual-level empowerment score, i.e., the weighted sum of her achievements across the six component indicators of the A-WEAI. In the second specification, we estimate separate yield equations for each of these six indicators. This allows us to identify the individual effect each indicator has on improving agricultural productivity. Five of the component indicators enter the yield equation as counts (the number of groups in which a woman is an active member, the number of decisions a woman makes about credit, the number of decisions a woman makes about production, the number of assets over which a woman has control, and the number of decisions a woman makes about household income). For *workload*, the sixth indicator, we created a dummy variable, which takes a value of 1 if the woman spent less than or equal to 10.5 hours of working on the day prior to the survey interview, and 0 otherwise. In both specifications, we estimate an additional yield function without including potentially endogenous input variables to check for robustness of the results.

We estimate the yield equations using a control function (CF) approach, which is more suitable for nonlinear models with endogenous variables [59] such as those in our case. As in other IV methods, the CF approach involves a two-stage estimation procedure such as two-stage least squares (2SLS) method, but it has the advantage of being able to estimate nonlinear models with endogenous regressors. The 2SLS method, for example, which estimates the first stage using the ordinary least square (OLS) method, cannot be used in our case because our first stage involves nonlinear models; although we use 2SLS to test the validity and relevance of our instruments as we are not aware of any other suitable IV diagnostic test method when the first stage is a non-linear model. Thus, in our first stage, women’s empowerment is predicted using the various instruments described in section 2.1 above, and the predicted values are included as covariates in the second stage. Our approach resembles the estimation procedures used in previous studies involving the WEAI [40, 41]. However, whereas these studies have tended to treat the WEAI as continuously distributed and estimate the first stage using OLS, we properly account for the bounded nature of the empowerment score and estimate the first stage using a fractional response Probit model (FRPM) [60, 61]. Failing to treat the empowerment score as bounded—as previous studies have done—and estimating the first stage using OLS may lead to inconsistent estimates and miss some potentially important nonlinearity. Following [62], therefore, we performed the regression specification error test (RESET) for functional misspecification in the first stage, and reject the null hypothesis that the equation has a linear functional form (F[3, 2432] = 2.96).

For the first-stage estimation of all the individual indicators of empowerment in agriculture except *workload*, we follow a similar CF approach as above, which again is justified by the nonlinear nature of these models, except that we use count regression models. Specifically, the *input in productive decisions*, *asset ownership* and *control over use of income* indicators are estimated using Poisson regression. The *group membership* indicator is estimated using zero-inflated Poisson because of excessive zeros. The indicator *access to and decisions about credit* is estimated using a negative binomial model due to over-dispersion. For *workload*, the first-stage regression is estimated using a Probit model. The yield equations are estimated using OLS.

## 3. Description of the study area, data and sample household characteristics

The data for this study come from western Kenya, where PPT was developed and tested, and where it is now being promoted to increase maize productivity through controlling *Striga* species (witchweed) and stemborers as well as improving soil fertility. Farmers in this region have two growing seasons: a long rainy season (March—August) and a short one (September—December). The household and individual survey data were collected by the International Centre of Insect Physiology and Ecology (icipe) between July and August 2016 for the 2015/16 cropping seasons while farmers were harvesting and threshing maize planted in 2016/17. In respect of harvesting and threshing, female invest substantially more labour than male in their households [10].

**Fig 1:**
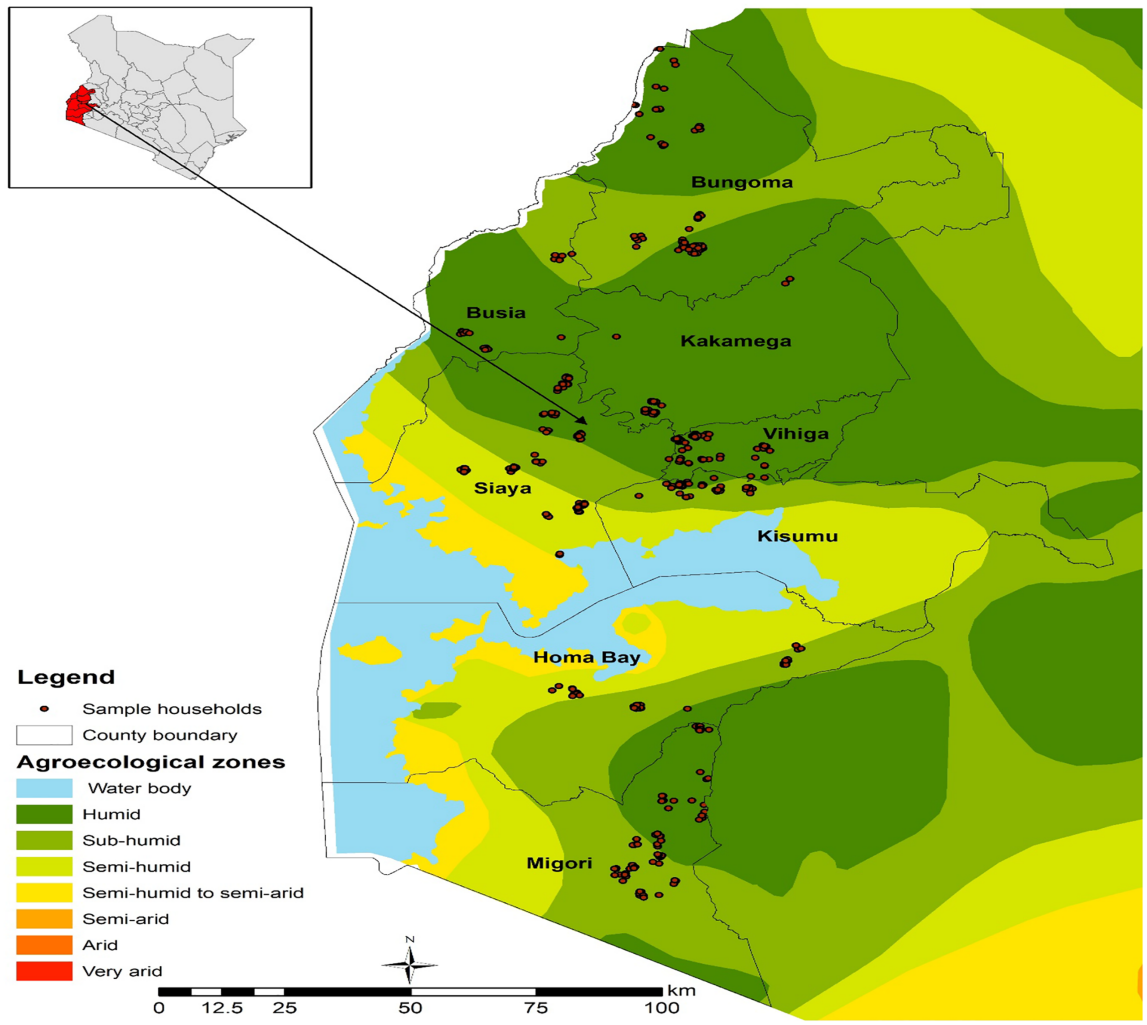
Study area and distribution of sample households.

Due to resource limitations we selected 9 of the 11 counties where PPT was being used by farmers. The selected counties were Bungoma, Busia, Homa Bay, Kakamega, Kisumu, Migori, Siaya, Trans Nzoia and Vihiga (Fig 1). Next, between 3 and 11 villages were randomly selected in each county using the probability-proportional-to-size sampling method. Within each village, between 2 and 21 households were randomly selected, also proportional to their size. In total, 60 villages and 711 farmers operating on 4,494 plots were surveyed. Of the total plots (4,346) cultivated by the households, 2,481 plot observations were planted with maize, which is the focus of this study. After dropping outlier observations and observations that had missing values for some variables, the usable sample amounted to 707 households and 2,248 maize plots.

Separate questionnaires were designed for households and individuals. The survey tool was administered using semi-structured interviews by trained enumerators who spoke and understood the local languages. Respondents’ participation in the survey was voluntary. The questionnaire had an introductory statement that sought the respondent’s consent to participate in the survey. While the household questionnaire was administered jointly to the principal male and female adult decision-makers in the household, the individual questionnaire was administered to the principal female decision-maker only, via a private interview conducted away from the male adult decision-maker to avoid data/response contamination. While both questionnaires aimed to capture gender dynamics within the household, the individual questionnaire was aimed specifically at eliciting the data required to compute the A-WEAI score for the adult female decision-maker. Due to budget constraints, the A-WEAI -oriented questionnaire was administered to women only, i.e., male adult decision-makers in a household were not interviewed.

The household questionnaire administered to both spouses elicited information on, among other things, household and individual demographic characteristics; crop and livestock production and utilisation data (input data, consumption data, marketing, etc.); ownership of productive assets (labour, land and livestock) by sex; farming practices such as PPT adoption; plot characteristics and management; access to development services (extension, credit and markets); non-agricultural income-generating activities; and social capital and network variables such as spouses’ membership of rural institutions. Table 3 offers definitions and summary statistics for all the variables used in the analysis.

**Table 3:** Description of variables and summary statistics.

Variables	Mean	Standard deviation
Outcome variable		
Maize yield (kg/acre)	1,123.33	872.31
Women’s empowerment indicators		
Women’s overall empowerment score (based on aggregate weighted score)	0.63	0.19
Number of production decisions in which the woman participates (out of 4)	1.95	0.86
Number of assets over which the woman has control (out of 7)	2.46	1.54
Number of credit-related decisions in which the woman participates (out of 12)	2.99	2.65
Number of income decisions in which the woman participates (out of 9)	4.84	2.08
Number of formal and informal groups to which the woman belongs (out of 10)	1.309	2.17
Time adequacy (1 = woman worked less than or equal to 10.5 hours, 0 = worked more than 10.5 hours)	0.27	0.45
Total workload (hours)	12.68	3.67
Domestic workload (hours)	7.01	4.62
Farm workload (hours)	3.81	2.77
Household socio-economic characteristics		
Sex of household head (1 = male, 0 = female)	0.69	0.46
Age of man (years)	55.24	11.70
Age of woman (years)	48.45	13.01
Formal education of man (years)	7.99	4.75
Formal education of woman (years)	7.62	3.62
Livestock ownership (TLUs)	1.99	2.45
Agricultural inputs and practices		
Fertiliser use (kg/acre)	61.75	150.13
Seeds and chemicals input (KSh/acre)	1,206.49	1678.02
Total labour input (person-days/acre)	83.56	91.37
Hired labour input (person-days/acre)	10.00	21.11
Plot intercropped (1 = Yes, 0 = No)	0.86	0.35
Crop rotation on plot (1 = Yes, 0 = No)	0.04	0.20
Push—pull technology used on plot (1 = yes, 0 = no)	0.30	0.46
Farmer has confidence in the skill of extension officers (1 = yes, 0 = no)	0.77	0.42
Credit-constrained household (1 = needed credit, but did not get it, 0 otherwise)	0.57	0.50
Plot-level attributes		
Distance from residence to plot (walking minutes)	4.00	11.17
Shallow depth plot (1 = yes, 0 = no)	0.06	0.24
Medium depth plot (1 = yes, 0 = no)	0.45	0.50
Deep depth plot (1 = yes, 0 = no)	0.49	0.50
Low soil fertility plot (1 = yes, 0 = no)	0.07	0.25
Medium soil fertility plot (1 = yes, 0 = no)	0.53	0.50
High soil fertility plot (1 = yes, 0 = no)	0.40	0.49
Gentle slope plot (1 = yes, 0 = no)	0.50	0.50
Medium slope plot (1 = yes, 0 = no)	0.48	0.50
Steep slope plot (1 = yes, 0 = no)	0.02	0.13
Plot tenure (1 = owned, 0 = rented)	0.92	0.28
Plot affected by pests and diseases (1 = yes, 0 = no)	0.48	0.50
Community-level variables		
Distance from residence to input supply shop (walking minutes)	51.49	43.36
Distance from residence to main market (walking minutes)	59.78	39.80
Distance from residence to agricultural extension office (walking minutes)	70.43	53.84
Dummy for sub-region (1 = Luo region, 0 = Luhya region)	0.65	0.48
Other variables		
Cropping season (1 = long rainy season, 0 = short rainy season)	0.53	0.50
Plot (and household) observations	2,436 (707)	

Source: *icipe* survey data

Note: Farm workload includes time spent on: [crop farming activities, livestock production, fishing and marketing]. Domestic workload includes time spent on: [cooking, fetching water, collecting firewood, child care and any another domestic work].

## 4. Results and discussion

### 4.1 Descriptive statistics

**Fig 2:**
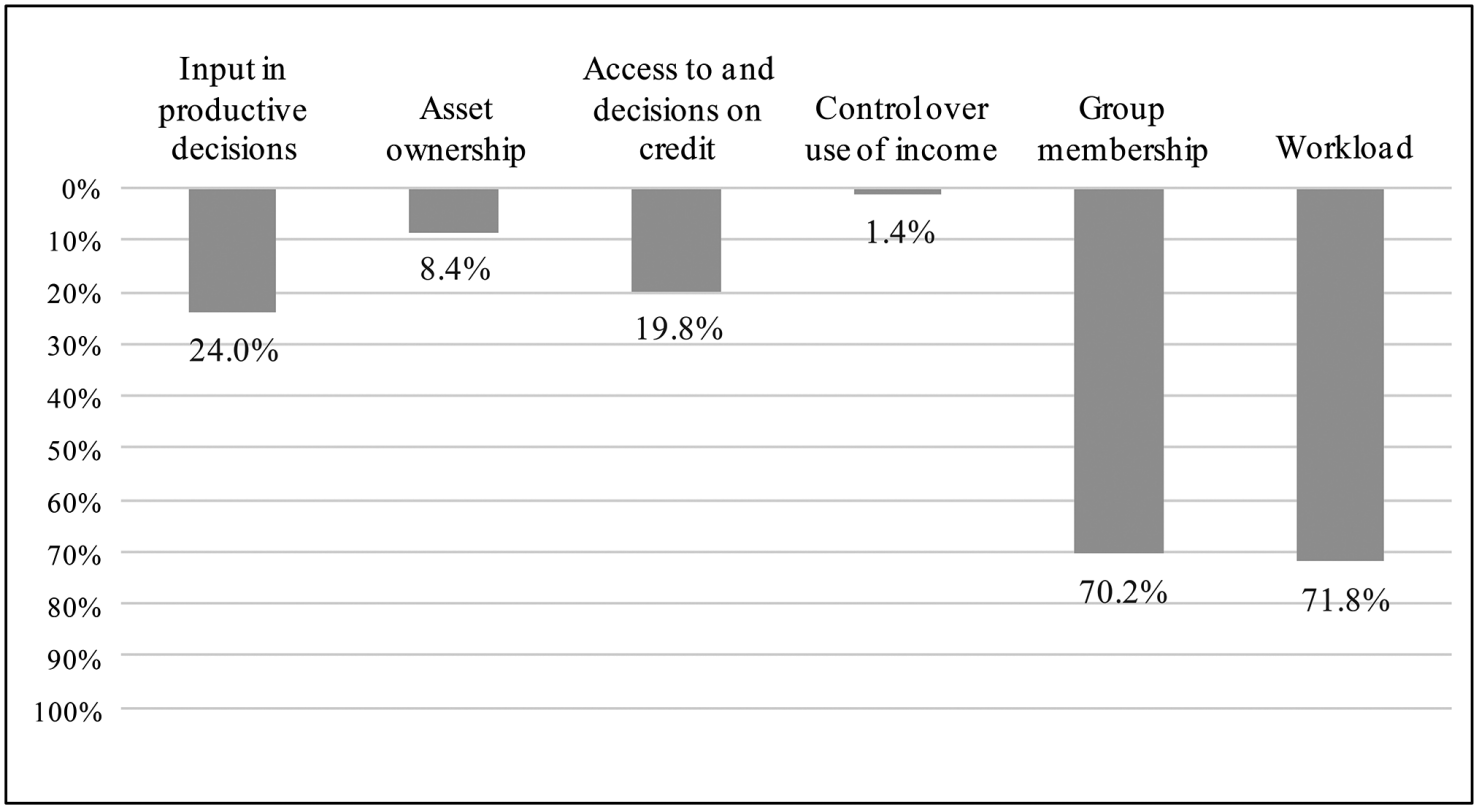
Percentage of women registering inadequacy in terms of each A-WEAI indicator.

The descriptive statistics show that, on average, women achieve adequacy in 64% of the weighted indicators in the A-WEAI. Each indicator takes a value of 1 if a woman achieves adequacy according to cut-offs defined by [28], and 0 otherwise. Per the 80% cut-off utilised in [28], 65.9% of women in the sample would be considered disempowered, which is close to the baseline WEAI reported by [63] for northern Kenya, where 68.4% of women in the area were reported as being disempowered. With respect to the individual indicators that comprise the A-WEAI, women were most likely to achieve adequacy in *asset ownership*, *access to and decisions on credit*, and *control over use of income*, and least likely to achieve adequacy in *group membership* and *workload* (see Fig 2). In households with both spouses present, the principal female decision-maker is about seven years younger than her male counterpart, on average, and has attained 0.36 fewer years of education.

Maize is the main staple food crop in western Kenya, providing both food and income to rural households in the area. The crop contributes about 68% of daily per capita cereal consumption, 35% of total dietary energy consumption and 32% of total protein consumption in the country [64]. Households in the sample reported maize harvests of 1,123 kg per acre (2.77MT/ha) on average. While western Kenya is part of a high potential maize production belt, the most important factor contributing to better yields may be the adoption of PPT by households in the study region. The average maize yield on PPT plots was 1,507 kg per acre (3.72 MT/ha), compared with an average on non-PPT plots of 960 kg per acre (2.37 MT/ha). Strengthening this finding is the fact that the sample households’ PPT yield is close to the on-farm yield 3.50 MT/ha reported by [58].

**Fig 3:**
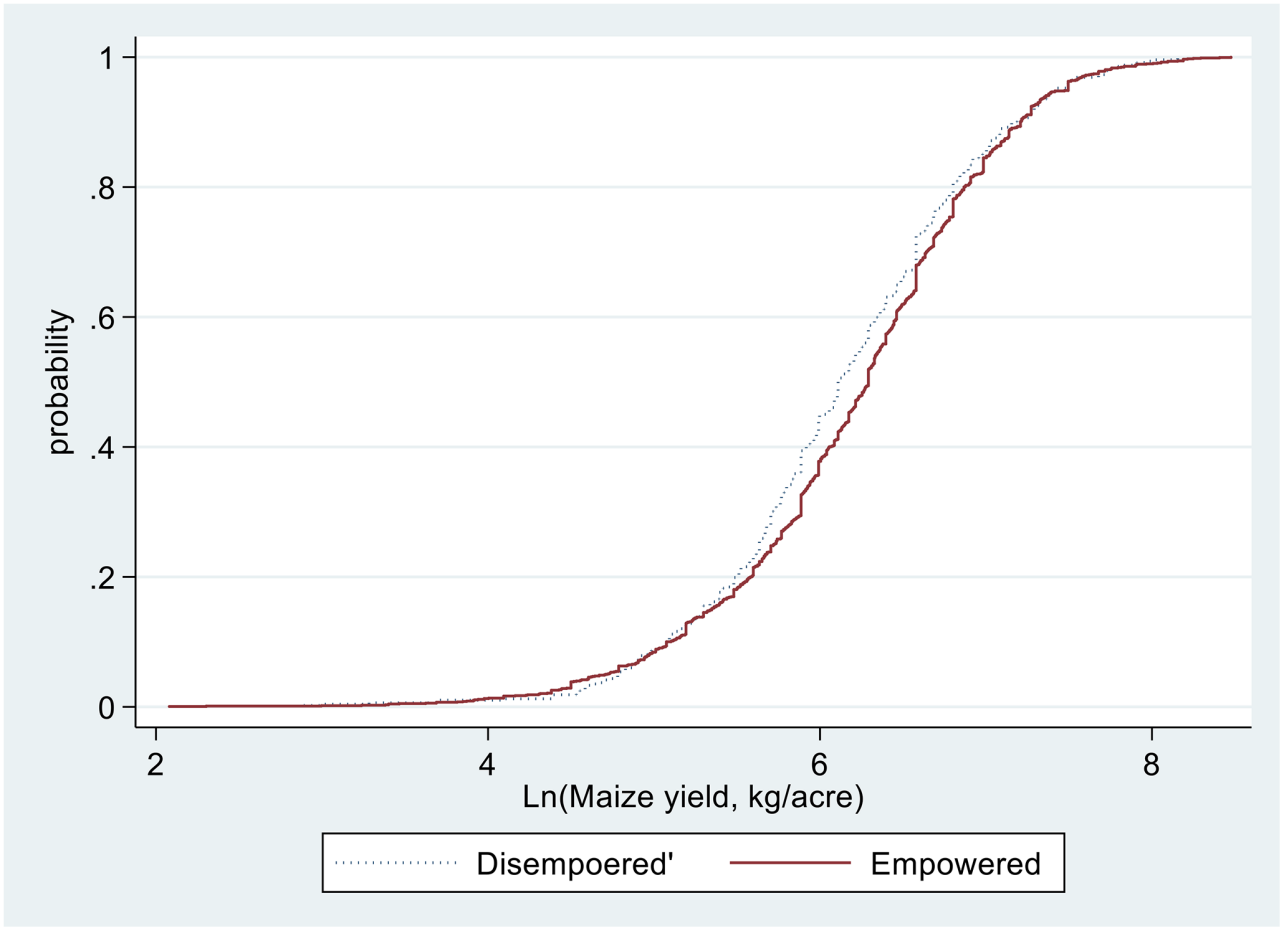
Distribution of maize yield.

Our study further reveals that the probability of adoption PPT is higher for households with empowered women (52%) compared with households with disempowered women (50%). A stochastic dominance analysis also shows that the maize yield distribution of households with empowered women in production decision appear to dominate that of households with disempowered women (Fig 3). The vertical distance between the cumulative density function of *empowered* and *disempowered* is significant at better than the 5% level. Although this should be subjected to rigorous analysis, the result is in line with the existing empirical literature where empowering women or closing gender gaps would lead to higher agricultural productivity [26, 43, 65].

**Fig 4:**
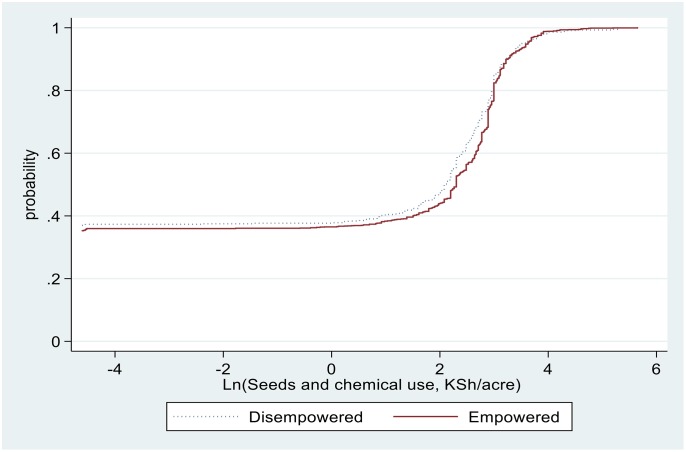
Distribution of seeds and pesticides expenditure in maize farming.

**Fig 5:**
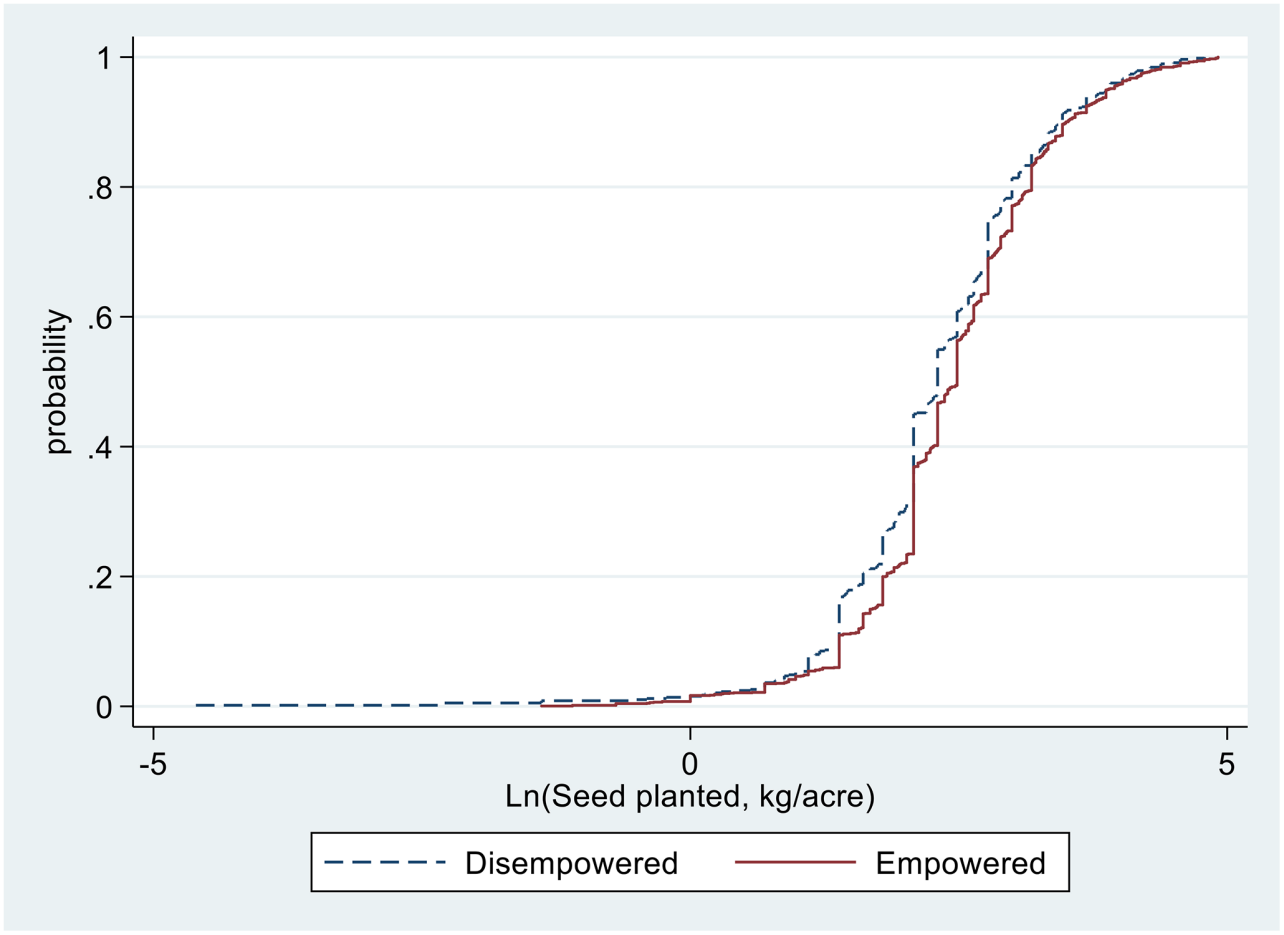
Distribution of seed planted in maize farming.

**Fig 6:**
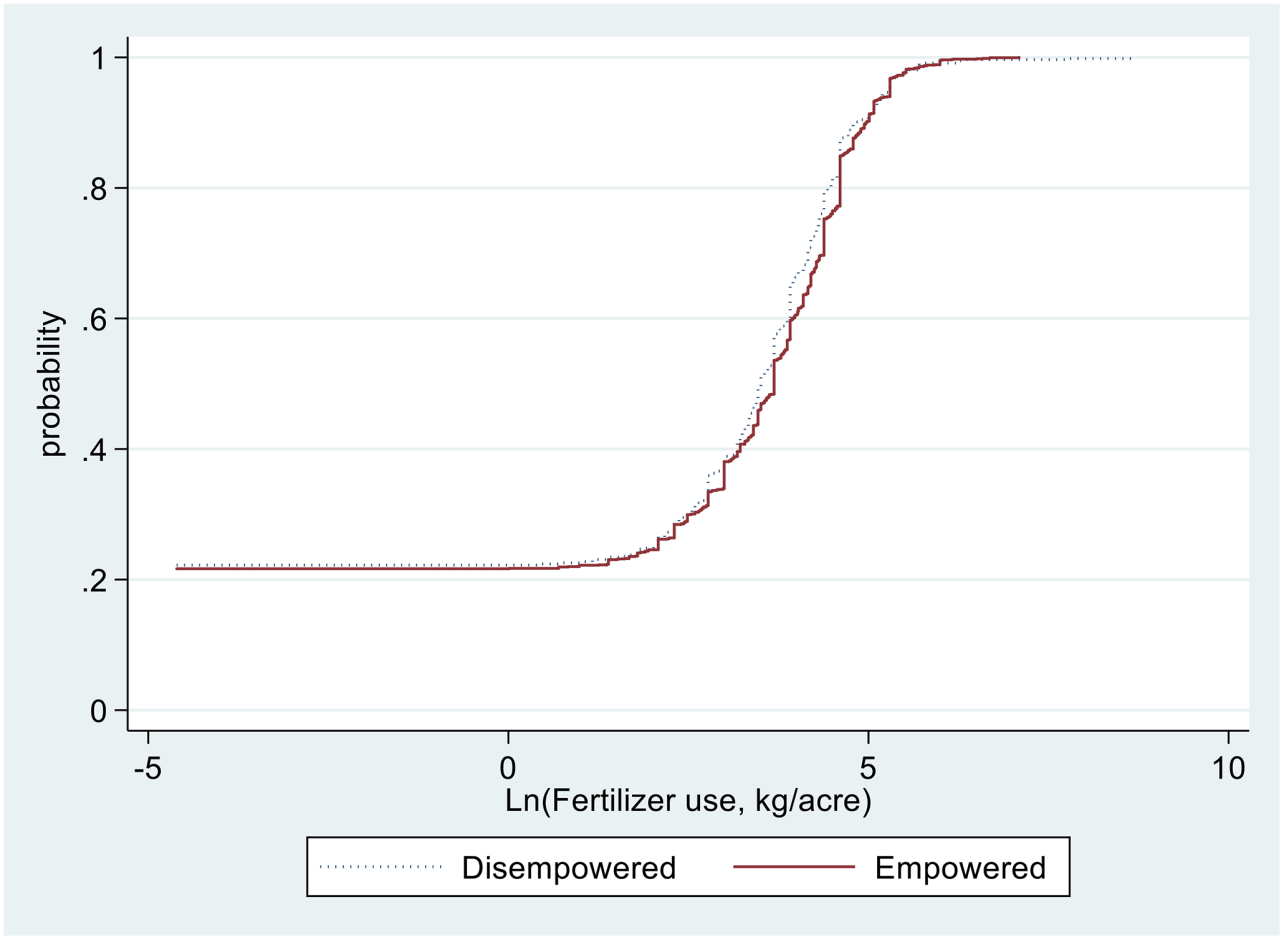
Distribution of fertiliser application rates in maize farming.

**Fig 7:**
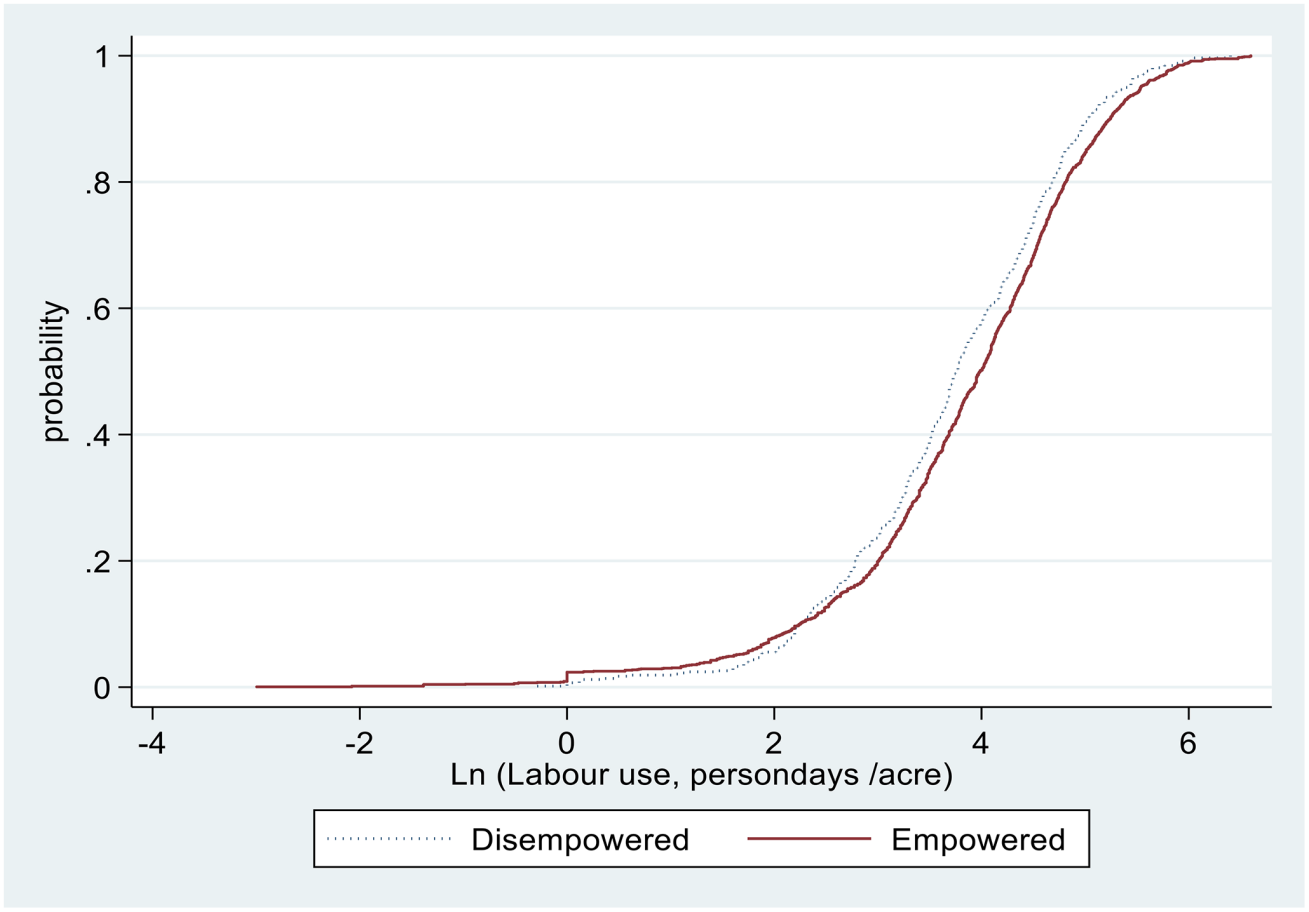
Distribution of labour use in maize farming.

The pathways for higher yields associated with households with empowered women could be due to a combination of factors, including increased use of improved technologies such as inorganic fertilisers, of agronomic practices such as PPT, and of pesticides and labour use. For example, as illustrated in Figs 4 to 7, sample maize farming households with empowered women appear to stochastically dominate those with disempowered women with respect to seeds and pesticides expenditure (Fig 4), amount of seed planted (Fig 5), fertiliser application rates (Fig 6), and labour allocation (Fig 7). Furthermore, adoption of PPT and improved maize varieties is higher for households with empowered women (52% and 72%, respectively) compared with those for disempowered women (46% and 68%, respectively).

### 4.2 Econometric results

Tables 4 and 5 present the results of the first-stage equations estimated using fractional response, count and Probit regression models along with diagnostic tests for the relevance of the IVs used in this study. Because our primary interest is on the impacts of empowerment, the first-stage regression estimates are not discussed. However, it is worth mentioning that the endogeneity test rejects the null hypothesis that overall women’s empowerment and the individual indicators of women’s empowerment are exogenous except for workload indicator. We also performed tests for under- and over-identification and reject the null hypothesis that the outcome regression models are under-identified, i.e., the instruments are relevant or correlated with endogenous regressors. However, we fail to reject the null hypothesis that the instruments are valid instruments (overidentification test), or uncorrelated with error terms and that the excluded instruments are correctly excluded from outcome equations.

**Table 4:** First-stage results for women’s overall empowerment score and IV diagnostics.

Variables	Coefficient
Difference in age between principal male and principal female in household (years)	-0.00
	(0.002)
Difference in education between principal male and principal female in household (years)	-0.01***
	(0.003)
Years of residence in village (women only)	0.00***
	(0.001)
Wife brought assets into marriage (1 = yes, 0 = no)?	0.03
	(0.024)
Proportion of household members below 5 years	-0.08
	(0.085)
Proportion of household members between 5 and 9 years	-0.01
	(0.079)
Proportion of household members between 10 and 14 years	0.04
	(0.081)
Proportion of household members between 15 and 19	0.12*
	(0.065)
Proportion of household members between 20 and 44	-0.16**
	(0.070)
Proportion of household members between 45 and 60	-0.09
	(0.067)
Other covariates	Yes
Sub-region dummy	Yes
Constant	0.75***
	(0.100)
Wald chi-square test	1291.07***
Observations	2,436
IV diagnostic tests	
Endogeneity test, H_0_: exogenous	3.95**
Over-identification test (Hansen J statistics), H_0_: instruments are valid	11.15
Weak identification test (Kleibergen-Paap rk Wald F statistic)	119.06 (it is greater than critical value (30.53) for one endogenous and 9 excluded instruments)
Anderson-Rubin Wald test, p-value (F statistics version)	2.17**
Anderson-Rubin Wald test (*χ*2 version)	19.75 (0.020)**
Under-identification test (Kleibergen-Paap rk LM statistic)	427.90***

Note: Robust standard errors in parentheses. The first stage is estimated using a fractional response probit model. The IV diagnostic is based on 2SLS as we are not aware of any IV diagnostic test that could be used when the first stage is nonlinear.

* p<0.1,

** p<0.05,

*** p<0.01.

**Table 5:** First-stage results for individual indicators of women’s empowerment and IV diagnostics.

Variables	Productive decisions	Asset ownership	Income decisions	Credit decisions	Group membership	Workload
Difference in age between principal male and principal female in household	-0.00	-0.01***	0.00*	-0.00	-0.07***	-0.02***
	(0.001)	(0.001)	(0.001)	(0.002)	(0.002)	(0.004)
Difference in education between principal male and principal female in household (years)	-0.01***	-0.04***	-0.01***	-0.04***	-0.15***	0.00
	(0.002)	(0.002)	(0.002)	(0.004)	(0.003)	(0.007)
Diversity of associations in village (Number of types of association)			0.05***	0.11***		
			(0.004)	(0.008)		
Wife brought assets into marriage? (1 = yes, 0 = no)	0.16***	0.16***	0.08***			
	(0.023)	(0.033)	(0.022)			
Proportion of household members below 5 years						-0.29
						(0.312)
Proportion of household members between 5 and 9 years	-	-	-	-	-	-0.34
	-	-	-	-	-	(0.291)
Proportion of household members between 10 and 14 years	-	-	-	-	-	-0.11
	-	-	-	-	-	(0.267)
Proportion of household members between 15 and 19 years	-	-	-	-	-	0.01
	-	-	-	-	-	(0.270)
Proportion of household members between 20 and 44 years	-	-	-	-	-	-0.51**
	-	-	-	-	-	(0.252)
Proportion of household members between 45 and 60 years	-	-	-	-	-	-0.75***
	-	-	-	-	-	(0.217)
Other covariates	Yes	Yes	Yes	Yes	Yes	Yes
County dummies	Yes	Yes	Yes	Yes	Yes	Yes
Constant	0.58***	1.25***	1.19***	-0.01	-0.01	1.31***
	(0.111)	(0.140)	(0.105)	(0.222)	(0.222)	(0.229)
Log likelihood	-3525.218	-4144.233	-5121.1461	-5320.448	-3405.94	-1209.07
Observations	2,436	2,436	2,436	2,436	2,436	2,436
IV diagnostic tests						
Endogeneity test, H_0_: exogenous	2.90*	0.68	4.74**	30.18***	4.05**	1.64
Over-identification test (Hansen J statistics)	0.78	0.093	0.667	4.12	0.37	10.90
Weak identification test (Kleibergen-Paap Wald rk F statistic)	32.18	97.98	16.05	81.04	476.04	5.62
Under-identification test (Kleibergen-Paap rk LM statistic)	82.88***	192.10***	43.69***	204.57***	521.57***	38.05***

Note: Robust standard errors in parentheses. The *input in productive decisions* and *asset ownership* indicators were estimated using Poisson regression. The *group* leadership indicator was estimated using the zero-inflated Poisson because of excessive zeros, while *access to and decisions on credit* was estimated using a negative binomial regression model due to over-dispersion. For *workload* we used the probit regression model. The IV diagnostic estimates are based on 2SLS.

* p<0.1,

** p<0.05,

*** p<0.01.

### 4.3 Impact of women’s empowerment on maize yields

Table 6 presents estimates of the effect of women’s overall empowerment scores on maize yield, as derived from the CF-FRPM approach as well as by using OLS and 2SLS estimation methods for comparison purposes. Because we use the predicted values of the endogenous variable in the second-stage estimation, we report bootstrapped standard errors for CF-FRPM to improve the efficiency of our estimates. The results are consistent across the different regression models and show that an increase in women’s overall empowerment score significantly improve maize yields, suggesting the importance of improving women’s empowerment in Kenya agriculture to reduce food insecurity and poverty. Inputs (fertiliser, value of seed and pesticides) and agricultural practices (adoption of PPT, intercropping and rotation) are less likely to correlate with errors of yield equation because many explanatory variables that influence the former variables are included. Also, the decision to use these variables (i.e. the input and agricultural practice) occurred before the maize harvest period. However, results excluding these variables in the regression models are qualitatively similar to including them in those models. For instance, an increase in women’s empowerment by 1% led to a 6.4% increase in maize yield when we used CF-FRPM, and 16.8% when we used 2SLS. Other significant determinants of maize yield in the study area include labour allocation, fertiliser application, expenditure on seed and pesticides, use of push—pull technology on a plot, intercropping, perceived incidence of pests and diseases on a plot, distance to input supply shops and output markets, sex of household head, and credit constraints (i.e., where a household needed but was unable to get credit) (Table 6).

**Table 6:** Effects of women’s overall empowerment on maize yield (Dependent variable—Ln(Maize yield, kg/acre)).

Variables	OLS	2SLS	CF-FRPM
Women’s overall empowerment score	0.16*	0.38**	0.13***
	(0.083)	(0.152)	(0.045)
Ln(Labour use, person-days/acre)	0.15***	0.15***	0.15***
	(0.014)	(0.014)	(0.015)
Ln(Seed and chemical use, KSh/acre)	0.03***	0.03***	0.03***
	(0.004)	(0.004)	(0.004)
Ln(Fertiliser use, kg/acre)	0.04***	0.04***	0.04***
	(0.004)	(0.004)	(0.005)
Push—pull technology on the plot	0.22***	0.21***	0.21***
	(0.038)	(0.038)	(0.034)
Plot intercropped	0.50***	0.51***	0.51***
	(0.041)	(0.041)	(0.040)
Crop rotation on plot	-0.00	0.00	0.01
	(0.017)	(0.017)	(0.016)
Ln(Distance from residence to plot)	-0.19***	-0.20***	-0.20***
	(0.032)	(0.032)	(0.029)
Plot affected by pests and diseases	0.19***	0.18***	0.18***
	(0.030)	(0.029)	(0.029)
Cropping season	-0.02***	-0.02***	-0.02***
	(0.007)	(0.007)	(0.007)
Livestock ownership	-0.09***	-0.09***	-0.09***
	(0.031)	(0.031)	(0.030)
Sex of household head	0.02	0.02	0.02
	(0.038)	(0.038)	(0.044)
Credit-constrained household	-0.06***	-0.07***	-0.06***
	(0.021)	(0.021)	(0.020)
Ln(Distance from residence to input supply shop)	0.06***	0.06***	0.06***
	(0.020)	(0.020)	(0.022)
Farmer has confidence in the skill of extension officers	0.05*	0.05**	0.05**
	(0.024)	(0.024)	(0.024)
Ln(Distance from residence to agricultural extension office)	-0.01	-0.01	-0.01
	(0.058)	(0.058)	(0.053)
Ln(Distance from residence to main market)	-0.00	-0.00	-0.00
	(0.052)	(0.052)	(0.049)
Plot tenure	-0.03	-0.04	-0.04
	(0.031)	(0.031)	(0.028)
Medium slope plot	-0.35***	-0.37***	-0.37***
	(0.112)	(0.111)	(0.134)
Steep slope plot	0.01	0.01	0.01
	(0.033)	(0.033)	(0.029)
Low soil fertility plot	-0.12*	-0.12*	-0.12**
	(0.063)	(0.064)	(0.055)
Medium soil fertility plot	-0.02	-0.03	-0.03
	(0.063)	(0.063)	(0.071)
Medium depth plot	-0.07	-0.09	-0.09
	(0.063)	(0.064)	(0.067)
Deep depth plot	-0.24***	-0.24***	-0.24***
	(0.035)	(0.035)	(0.033)
County dummies	0.16*	0.38**	0.13***
	(0.083)	(0.152)	(0.045)
Constant	5.54***	5.39***	5.58***
	(0.170)	(0.191)	(0.145)
R-squared	0.29	0.28	0.29
Wald Chi2/F test	53.02***	52.59***	2609.01***
Observations	2,436	2,436	2,436

Note: Bootstrapped errors for CF-FRPM and robust standard errors for OLS and 2SLS models in parentheses.

* p<0.1,

** p<0.05,

*** p<0.01.

### 4.4 Dimensions of women’s empowerment that matter most in increasing maize yields

Table 7 presents estimates of the effects of each individual indicator of women’s empowerment on maize yields, estimated using a similar approach as in section 4.3 above (The results reported in Tables 6 and 7 are robust to the exclusion of input variables from the yield equation). The results indicate that all six indicators of women’s empowerment except *workload* are positively and significantly associated with maize yields. We note considerable heterogeneity in the magnitude of each indicator’s effect on maize yields. Thus, while all indicators of women’s empowerment are important, number of production decisions indicator has greater effect on improving agricultural productivity. If the number of production decisions made by women increases by one-unit, maize productivity can increase by 32 percent (This is computed based on 100 × *e*^*β*^ − 1, as we log-level regression specification). The same values for income, group membership and assets indicators are 14, 15 and 13 percent, respectively. Although not reported, qualitatively comparable results found using the two stages-least square (2SLS). Except the *workload* indicator, all other indicators have a positive and significant effects on maize yields.

**Table 7:** Effects of individual indicators of women’s empowerment on maize yield (Dependent variable—Ln(Maize yield, kg/acre)).

Variables	Production decisions	Asset ownership	Income decisions	Credit decisions	Group membership	Workload
Number of production decisions in which the woman participates	0.28***					
	(0.087)					
Number of assets over which the woman has control		0.12***				
		(0.027)				
Number of income decisions in which the woman participates			0.13***			
			(0.027)			
Number of credit-related decisions in which the woman participates				0.08***		
				(0.016)		
Number of formal and informal groups to which the woman belongs					0.14*	
					(0.079)	
Time adequacy						-0.08
						(0.140)
Ln(Labour use, person-days/acre)	0.14***	0.15***	0.12***	0.14***	0.14***	0.14***
	(0.011)	(0.016)	(0.014)	(0.011)	(0.012)	(0.015)
Ln(Seed and chemical use, KSh/acre)	0.02***	0.03***	0.02***	0.03***	0.03***	0.03***
	(0.005)	(0.004)	(0.004)	(0.004)	(0.005)	(0.004)
Ln(Fertiliser use, kg/acre)	0.04***	0.04***	0.04***	0.04***	0.04***	0.04***
	(0.005)	(0.004)	(0.006)	(0.004)	(0.005)	(0.006)
Push—pull technology on a plot	0.20***	0.19***	0.20***	0.17***	0.21***	0.22***
	(0.041)	(0.043)	(0.031)	(0.038)	(0.037)	(0.039)
Plot intercropped	0.48***	0.52***	0.49***	0.54***	0.51***	0.50***
	(0.041)	(0.033)	(0.038)	(0.041)	(0.044)	(0.040)
Crop rotation on plot	0.00	0.00	-0.02	0.01	0.00	-0.01
	(0.021)	(0.021)	(0.020)	(0.018)	(0.017)	(0.017)
Ln(Distance of from residence to plot)	-0.25***	-0.22***	-0.24***	-0.21***	-0.20***	-0.19***
	(0.042)	(0.032)	(0.026)	(0.031)	(0.031)	(0.034)
Plot affected by pests and diseases	0.19***	0.18***	0.20***	0.18***	0.19***	0.19***
	(0.034)	(0.037)	(0.026)	(0.033)	(0.034)	(0.033)
Cropping season	-0.01*	0.00	-0.02***	-0.02***	-0.04***	-0.02***
	(0.007)	(0.011)	(0.006)	(0.007)	(0.010)	(0.006)
Livestock ownership	-0.09***	-0.11***	-0.09***	-0.08**	-0.07*	-0.09**
	(0.028)	(0.025)	(0.029)	(0.033)	(0.039)	(0.036)
Sex of household head	0.06	0.04	0.02	0.06**	-0.01	0.02
	(0.047)	(0.032)	(0.036)	(0.030)	(0.044)	(0.034)
Credit-constrained household	-0.07***	-0.07***	-0.05**	-0.07***	-0.06**	-0.06***
	(0.019)	(0.019)	(0.022)	(0.019)	(0.022)	(0.020)
Farmer has confidence in the skill of extension officers	0.06***	0.06***	0.08***	0.04*	0.05***	0.06**
	(0.020)	(0.020)	(0.018)	(0.023)	(0.020)	(0.023)
Ln(Distance from residence to input shop)	0.07***	0.07***	0.05**	0.06***	0.06**	0.04**
	(0.022)	(0.023)	(0.023)	(0.022)	(0.026)	(0.021)
Ln(Distance from residence to extension office)	-0.04	-0.01	-0.03	-0.09	0.01	0.00
	(0.058)	(0.056)	(0.063)	(0.070)	(0.055)	(0.066)
Ln(Distance from residence to main market)	-0.01	0.03	0.02	-0.04	-0.01	0.00
	(0.056)	(0.050)	(0.057)	(0.046)	(0.053)	(0.061)
Plot tenure	-0.04	-0.01	-0.01	-0.04	-0.02	-0.02
	(0.029)	(0.031)	(0.029)	(0.029)	(0.029)	(0.032)
Medium slope plot	-0.40***	-0.33***	-0.36***	-0.33**	-0.37***	-0.34***
	(0.111)	(0.102)	(0.108)	(0.130)	(0.103)	(0.107)
Steep slope plot	0.03	-0.02	0.01	-0.02	0.02	0.01
	(0.032)	(0.042)	(0.027)	(0.030)	(0.032)	(0.038)
Medium soil fertility plot	-0.08	-0.10	-0.06	-0.11	-0.12*	-0.11**
	(0.067)	(0.069)	(0.076)	(0.069)	(0.066)	(0.053)
Low soil fertility plot	-0.09	-0.01	-0.13*	-0.07	-0.05	-0.01
	(0.063)	(0.063)	(0.070)	(0.068)	(0.061)	(0.078)
Medium depth plot	-0.10	-0.05	-0.11*	-0.11*	-0.08	-0.04
	(0.070)	(0.060)	(0.059)	(0.061)	(0.057)	(0.075)
Deep depth plot	-0.19***	-0.18***	-0.29***	-0.19***	-0.23***	-0.23***
	(0.039)	(0.037)	(0.037)	(0.035)	(0.036)	(0.036)
Sub-region dummy	5.13***	5.21***	5.12***	5.54***	5.39***	5.65***
Constant	(0.230)	(0.200)	(0.164)	(0.156)	(0.201)	(0.161)
	0.14***	0.15***	0.12***	0.14***	0.14***	0.14***
Wald chi-square test	4238.25***	2144.91***	2258.24***	3903.21***	1609.32***	3424.67***
R-squared	0.290	0.292	0.293	0.294	0.287	0.286
Observations	2,436	2,436	2,436	2,436	2,436	2,436

Note: Bootstrapped standard errors in parentheses.

* p<0.1,

** p<0.05,

*** p<0.01.

Other factor influencing maize yields include labour, fertiliser and seed and chemical use, use of PPT, intercropping, pest and disease occurrence, livestock ownership, credit constraints, extension services, sex of household head, and distance to input supply shops and output markets.

### 4.5 Effect of women’s empowerment on the productivity of female-managed, male-managed and jointly-managed plots

Table 8 presents estimates, using the CF approach, of the effects of women’s empowerment scores on maize yields according to whether plots were managed jointly by the principal female and male decision-makers, or managed solely by either one of them. The plot manager variables (*female-managed plots*, *male-managed plots* and *jointly-managed plots*) were created based on two questions: “Who in the household makes decisions on crops to be planted, input use and timing of crop activities?” and “Who in the household manages the plot?” The results reveal considerable heterogeneity in the effects of women’s empowerment on maize yields across the three types of plot management. More specifically, we find that the increased maize yields due to higher empowerment scores for the women were statistically significant for female-managed and male-managed plots.

**Table 8:** Effects of women’s overall empowerment on maize yield according to plot manager (Dependent variable—Ln(Maize yield, kg/acre)).

Variables	Female-managed plots	Male- managed plots	Jointly- managed plots
Women’s overall empowerment	0.98***	0.30**	-0.20
	(0.372)	(0.139)	(0.265)
Ln(Labour use, person-days/acre)	0.16***	0.15***	0.11***
	(0.041)	(0.023)	(0.030)
Ln(Seed and chemical use, KSh/acre)	0.04***	0.02***	0.04***
	(0.015)	(0.007)	(0.009)
Ln(Fertiliser use, kg/acre)	0.08***	0.05***	0.04***
	(0.015)	(0.007)	(0.010)
Push—pull technology on the plot	0.31***	0.16***	0.23***
	(0.120)	(0.060)	(0.084)
Plot intercropped	0.44***	0.56***	0.39***
	(0.128)	(0.075)	(0.094)
Crop rotation on plot	0.10**	0.04*	-0.12**
	(0.041)	(0.026)	(0.049)
Ln(Distance from residence to plot))	-0.38***	-0.18***	-0.28***
	(0.109)	(0.051)	(0.066)
Plot affected by pests and diseases	0.25***	0.16***	0.15**
	(0.086)	(0.046)	(0.066)
Cropping season	0.01	-0.00	-0.03*
	(0.020)	(0.014)	(0.016)
Livestock ownership	0.17*	-0.18***	-0.04
	(0.089)	(0.052)	(0.086)
Sex of household head	-0.04	-0.09	0.06
	(0.122)	(0.073)	(0.075)
Credit-constrained household	0.03	-0.12***	0.14***
	(0.069)	(0.035)	(0.053)
Farmer has confidence in the skill of extension officers	-0.08	0.11***	-0.05
	(0.059)	(0.033)	(0.045)
Ln(Distance from residence to input shop)	0.12	0.03	0.00
	(0.082)	(0.038)	(0.059)
Ln(Distance from residence to extension office)	-0.19	-0.03	0.02
	(0.151)	(0.096)	(0.104)
Ln(Distance from residence to main market)	-0.15	-0.03	0.05
	(0.151)	(0.080)	(0.134)
Plot tenure	0.22**	-0.08	0.05
	(0.095)	(0.051)	(0.065)
Medium slope	-0.63	-0.33*	-0.68***
	(0.412)	(0.171)	(0.252)
Steep slope	0.32***	0.03	-0.12*
	(0.109)	(0.053)	(0.071)
Low soil fertility plot	0.24	0.04	0.07
	(0.182)	(0.103)	(0.134)
Medium soil fertility plot	-0.02	0.03	-0.18
	(0.238)	(0.125)	(0.117)
Medium depth plot	-0.08	0.04	-0.12
	(0.238)	(0.124)	(0.133)
Deep depth plot	-0.29**	-0.37***	-0.14*
	(0.115)	(0.059)	(0.075)
Sub-region dummy	4.64***	5.66***	5.93***
Constant	(0.436)	(0.281)	(0.437)
R-squared	0.48	0.27	0.36
Observations	247	962	460

Note: Bootstrapped standard errors in parentheses.

* p<0.1,

** p<0.05,

*** p<0.01.

In Table 9 we compare selected attributes of female-, male- and jointly-managed plots to highlight some of the factors that may be critical to closing the gender gap in maize productivity. For example, female-managed plots tend to be less fertile and receive a lower intensity of fertilisers relative to the other plot-manager categories. Most notably, however, stark differences exist in the quantity of labour supplied to female-managed plots, relative to male-managed and jointly-managed ones: on average, female-managed plots receive roughly 32 fewer person-days per acre of total labour compared with their male-managed counterparts, and nearly 38 fewer person-days per acre than jointly-managed plots do.

**Table 9:** Selected attributes of plots managed by females only, males only and jointly by males and females.

Variable	Female-managed plots		Male-managed plots		Jointly-managed plots	
	Mean	Standard deviation	Mean	Standard deviation	Mean	Standard deviation
Plot-level attributes						
Size of plot (acres)	0.606	0.616	0.585	0.716	0.753	0.802
High soil fertility plot (1 = yes, 0 = no)	0.380	0.485	0.463	0.499	0.398	0.490
Medium soil fertility plot (1 = yes, 0 = no)	0.514	0.500	0.481	0.500	0.533	0.499
Low soil fertility plot (1 = yes, 0 = no)	0.109	0.313	0.056	0.230	0.070	0.255
Plot management practices						
Grain legume intercrops (1 = yes, 0 = no)	0.429	0.496	0.421	0.494	0.422	0.494
Fertilisers applied (1 = yes, 0 = no)	0.765	0.425	0.791	0.407	0.767	0.423
Quantity of fertiliser used (kg/acre)	56.44	67.97	59.06	69.02	56.03	36.019
Planted improved maize seed	0.648	0.479	0.626	0.484	0.670	0.471
Total labour use (person-days/acre)	49.90	48.15	81.44	69.94	87.94	72.56
Head of household is male	0.850	0.358	0.619	0.486	0.998	0.047
Number of observations	247		962		460	

Several possible explanations exist for this trend. Firstly, given the typical division of labour within SSA households and the heavy domestic workload this places on women, women may have less time than men to devote to cultivating their plots [2, 24]. Indeed, as reported earlier (Fig 2), more than 70% of women in the sample fail to achieve adequacy in the *workload* indicator, i.e., they spent more than 10.5 hours in paid and domestic work during the previous day. Secondly, restrictions on women’s mobility—due partly to this heavy burden of labour and partly to prevailing gender norms—may not only prevent them from accessing traditional markets to hire additional labour but could also make it more difficult for them to supervise any workers they did manage to hire.

## 5. Conclusion and policy implications

Women’s empowerment is widely perceived to be a key factor in closing gender gaps in agricultural productivity. In this paper, we explore the relationship between women’s empowerment in agriculture—measured using indicators derived from the A-WEAI—and maize productivity, using smallholder maize farmers in western Kenya as a case study. Controlling for potential endogeneity, we find that women’s empowerment leads to increased maize productivity, with the greatest gains derived from increases in women’s participation in decision-making on agricultural production.

Extending our analysis, we find evidence of heterogeneity in the effects of women’s empowerment on maize yields, namely that female- and male-managed plots experience significant improvements in yield. The effects of women’s empowerment on maize yields are insignificant for jointly-managed plots. Hence, our findings provide an important piece of evidence showing that women’s empowerment may contribute to closing the gender productivity gap.

We contribute to the gender and agriculture literature on two fronts. First, we provide direct evidence that women’s empowerment can contribute to closing the gender gap in agricultural productivity that has been widely observed in SSA [12, 66], and more generally, that improvements in women’s bargaining position may lead to more optimal allocations of the household’s productive resources, evidenced by higher productivity. Second, we illustrate how failing to correctly account for the bounded nature of the empowerment score may lead to overestimating the true impact of women’s empowerment on agricultural productivity. Furthermore, we demonstrate how to correct for this feature of the WEAI using various econometric procedures.

Our results also offer encouragement with respect to the effectiveness of policies and strategic interventions aimed at stimulating increased agricultural productivity in Kenya through women’s empowerment. Although we find that having the power to make important decisions about agricultural production to be the most important driver of maize productivity among the six indicators of women’s empowerment we tested, all except for the *workload* indicator had a significant effect on maize productivity. This speaks to the wide range of ways in which women’s empowerment impacts positively on agricultural productivity and suggests a great scope of possible interventions, ranging from financial inclusion mechanisms such as digital savings accounts, affordable mobile-money-based credit schemes and asset-building mechanisms, to programmes facilitating the formation of strong community associations for women.

In conclusion, while our study points towards women’s empowerment having a positive effect on maize yield, the cross-sectional nature of our data does not support an examination of the dynamic impacts associated with women’s empowerment and maize yield. Furthermore, our data are not nationally representative and, thus, may not reflect women’s empowerment status across Kenya. More research, using nationally representative and repeated data from Kenya and elsewhere in SSA, is needed to fully understand the relationship between women’s empowerment and maize yield.

## Supporting information

S1 Genderdata (DTA)
